# Characterization of Micronutrients, Bioaccessibility and Antioxidant Activity of Prickly Pear Cladodes as Functional Ingredient

**DOI:** 10.3390/molecules25092176

**Published:** 2020-05-06

**Authors:** Meriam Missaoui, Isabella D’Antuono, Massimiliano D’Imperio, Vito Linsalata, Sadok Boukhchina, Antonio F. Logrieco, Angela Cardinali

**Affiliations:** 1 Laboratory of Neurophysiology, Cellular Physiopathology and Biomolecules Valorization, Faculty of Science of Tunis, University Tunis El Manar, LR18ES03, Tunis 2092, Tunisia; meriam.missaoui@fst.utm.tn (M.M.); sadok.boukhchina@fst.rnu.tn (S.B.); 2 Institute of Sciences of Food Production (ISPA), National Council of Research (CNR), 70126 Bari, Italy; massimiliano.dimperio@ispa.cnr.it (M.D.); vito.linsalata@ispa.cnr.it (V.L.); antonio.logrieco@ispa.cnr.it (A.F.L.); angela.cardinali@ispa.cnr.it (A.C.)

**Keywords:** *Opuntia ficus indica*, polyphenols, in vitro digestion, cations, dietary fibre

## Abstract

The *Opuntia ficus indica* (L.) (OFI) is used as a nutritional and pharmaceutical agent in various dietary and value added products. This study underlines the possible use of native prickly pear cladode powder as a functional ingredient for health-promoting food production. To summarise, chemical characterization of polyphenols, minerals and soluble dietary fibre was performed; furthermore, the antioxidant activity and bioaccessibility of polyphenols and minerals were assessed. Eleven compounds between phenolic acids and flavonoids were identified, with piscidic acid and isorhamnetin derivatives being the most abundant. *Opuntia’s* dietary fibre was mainly constituted of mucilage and pectin, and was composed of arabinose, galactose, glucose, mannose, rhamnose, and xylose sugars. The polyphenols’ bioaccessibility was very high: piscidic acid at 200%, eucomic and ferulic acids >110% and flavonoids from 89% to 100%. The prickly pear cladode powder is also a source of minerals, as cations (calcium, sodium, potassium and magnesium) and anions (sulphate and chloride), with high magnesium bioaccessibilty (93%). OFI powder showed good capacity of radical scavenging measured by DPPH and ABTS methods, with 740 and 775 μmol Trolox/100 g OFI, respectively. Finally, the presented results allow the consideration of this natural product as a source of several essential nutrients, with a possible use in the food industry as a functional ingredient.

## 1. Introduction

The *Opuntia ficus indica* (L.) Miller (OFI), also known as the nopal cactus or prickly pear, is a dicotyledonous angiosperm plant originally from South America. It belongs to the *Cactaceae* family, which contains about 130 genera and nearly 1500 species [1]. This plant can grow in arid and semi-arid climates all over the world with great economic potential. In particular, about 70,000 ha have been devoted to OFI cultivation in Tunisia, 100,000 ha in Italy and 300,000 ha in Brazil, in addition to large areas in Algeria, Argentina, Chile, Mexico, and South and North Africa [2]. *Opuntia* produces edible stems known cladodes that replace leaves in their photosynthetic function; the cladodes’ interest has increased since the mid-70s. In the Mediterranean basin, OFI is mainly used as a food plant for its edible fruit and for animal feed supplementation [3,4]. In North Africa, instead, the cultivation is used to counteract soil erosion in arid areas and to substitute forage during drought [5]. The OFI cladodes have been used for centuries for nutritional and medical purposes, and their nutritional properties have recently been clarified by several scientific studies, as widely reported by different authors [6]. The industrial applications of OFI cladodes are mainly associated with the food industry for the preparation of juices, beverages, jams, sweeteners and tea, as well as agrochemicals, cosmetics, wastewater treatments [7,8,9] and in traditional medicine for the treatment of some chronic diseases [10,11]. Furthermore, OFI cladodes are also a source of carbohydrates and fibre, particularly pectin, lignin, mucilage, cellulose and hemicellulose, recognized for their positive influence on glucose and lipids metabolism, obesity control and for the prebiotic function for large intestine microbiota [12,13]. They are also recognized for the presence of bioactive compounds, flavonoids and phenolic acids among them, and hydroxycinnamic acids (piscidic and eucomic acids), rarely encountered in nature and restricted to plants exhibiting crassulacean acid metabolism and succulence [14,15,16,17]. Further, OFI cladodes present high values of important nutrients like minerals and vitamins which are able to regulate osteoporosis diseases [18]. Using diffractive microscopy and spectrophotometric techniques, Contreras-Padilla et al. have demonstrated that *Opuntia* cladodes are also able to bioaccumulate inorganic constituents such as magnesium oxide, calcium carbonate (calcite), calcium oxalate monohydrate, calcium-magnesium bicarbonate, potassium chloride and potassium peroxy-diphosphate [19]. However, OFI’s chemical composition can change with species, cultivar, physiological stage, environmental conditions and processing [16,20,21,22]. Regarding the latter, high temperature treatments (65 °C and 5 ms^−1^ airflow) affect the cladodes’ bioactive components (phenolic acid, flavonoid, ascorbic acid and β-carotene) influencing their stability which, otherwise, is preserved by mild temperature treatments (45 °C and 3 ms^−1^ airflow) [23]. The main objective of the presented paper is to valorize an ingredient obtained by mild technologies from OFI cladodes to use for the enrichment of widely consumed foods, such as bread, pasta and biscuits, for a possible functional food industrial application. To this purpose, the polyphenols profile, polysaccharides characterization, ions composition and antioxidant activity (as ABTS and DPPH radical scavengers) in dehydrated OFI cladodes were performed. Moreover, considering the influence of other plants’ cellular components on polyphenols and their fate during human digestion, a simulated gastrointestinal digestion was also executed. Further, this method allowed us to evaluate the cations’ bioaccessibility, an important point for assessing the health impact of a functional ingredient.

## 2. Results and Discussion

### 2.1. Bioactive Compounds in OFI Cladodes: Polyphenols, Dietary Fibre, Minerals

#### 2.1.1. Polyphenols Characterization

**Figure 1:**
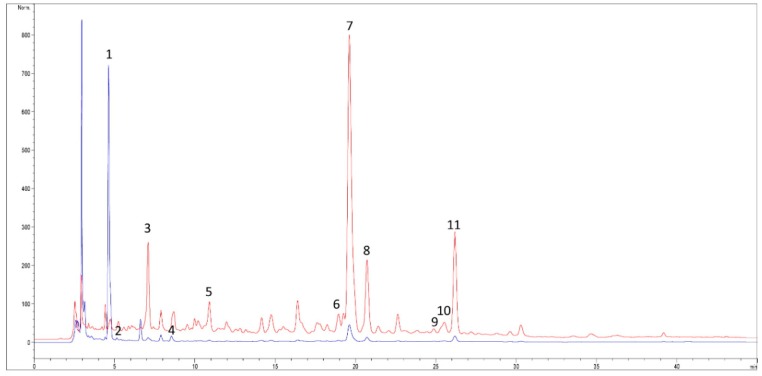
HPLC-DAD profile of *Opuntia ficus indica* cladodes (OFI) at two wavelengths: 280 nm (blue trace) and 325 nm (red trace). Polyphenol identification: 1 = piscidic acid I; 2 = piscidic acid II; 3 = ferulic acid derivative I; 4 = eucomic acid; 5 = ferulic acid derivative II; 6 = kaempferol derivative I; 7 = isorhamnetin derivative I; 8 = isorhamnetin derivative II; 9 = kaempferol derivative II; 10 = isorhamnetin derivative III; 11 = isorhamnetin 3-O-rutinoside (narcissin).

*Opuntia ficus indica* powder was characterized for its polyphenol content by HPLC with Diode Array Detector (HPLC-DAD); the results are shown in Table 1 and Figure 1.

From the analysis, 11 compounds between phenolic acids and flavonoids were identified. In particular, piscidic acid (PI) and isorhamnetin derivatives were the most abundant, followed by eucomic acid (EU), which was already identified in *Opuntia ficus indica* extract by Ginestra et al. [24]. According to the authors, PI represents almost 70% of the total polyphenols identified in OFI. This result is different from the study of De Santiago, et al. [25] which identified EU as the most abundant (50−60% total phenolic acids). In this study, both the PI and EU were quantified as tyrosol equivalent for the similar UV spectral data. Among the isorhamnetin derivatives, the only compound identified using the pure standard was isorhamnetin 3-O-rutinoside (narcissin); the other two major isorhamnetin derivatives (I and II) were probably isorhamnetin rutinoside–ramnoside and isorhamnetin hexoside–pentoside, according to results reported by De Santiago et al. [25]. Finally, smaller concentrations of kaempferol derivatives and ferulic acid derivatives were also detected according to the same authors [25].

#### 2.1.2. Dietary Fibre Determination

The water-soluble polysaccharide fraction that occurs in OFI cladodes was recovered following two different extraction procedures. In particular, the mucilage (MF) was extracted with hot water; subsequently, a conventional method for pectin isolation (PF), i.e., water-acid extraction, was performed on the pellet [26,27]. Finally, on the OFI powder without mucilage removal, an acid extraction process was applied to recover the pectin and mucilage fraction (PMF). The obtained results (Table 2) showed that the MF had the highest extraction yields, expressed as g/100 g of OFI, followed by PMF and PF.

**Figure 2:**
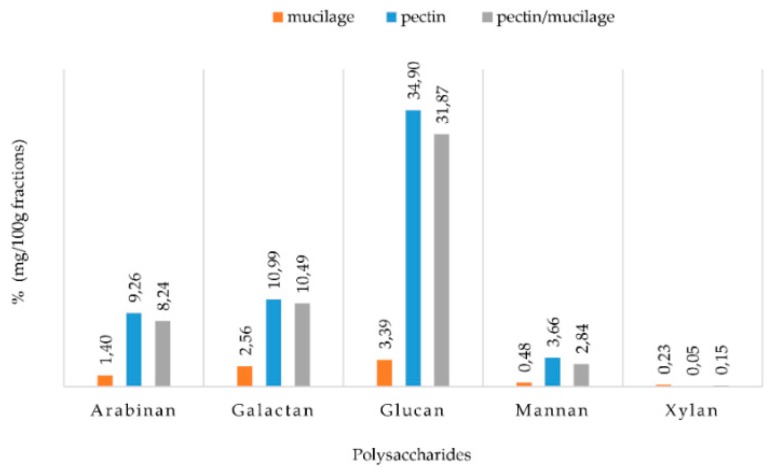
Sugar contents of three water-soluble fibre fractions extracted from *Opuntia ficus indica* cladode powder (OFI).

Although some differences related to the cultivar, the physiological state of cladodes and to the extraction method could be found, these results confirm that mucilage is the most important polysaccharide fraction of the cladodes [28]. When, conversely, the total sugars present in the individual fractions are considered, it is possible to observe that the pectin fraction had the highest sugar concentration (66.4%). These results were in agreement with Bayar et al. [26], which found different functional properties for the two fractions: foaming and emulsifying characteristics for pectin, and water and oil holding capacities for mucilage, highlighting the potential application of these fractions as natural additives in the food industry. The carbohydrate analysis of the natural sugars, following three step acid hydrolysis, showed the presence of arabinose, galactose, glucose, mannose, rhamnose and xylose. Their amount, expressed as concentrations of polymeric sugars, is shown in the Figure 2.

The pectin fraction presented the highest sugar content, with glucan as the most abundant estimated polymer (35%), followed by galactan (11%), arabinan (9%), mannan (4%) and a very small amount of xylan. The composition of the disaccharides in the PMF was very similar to what was discussed for PF. Meanwhile, the MF, although its extraction yield was the highest (18.5%), contained the lowest concentration of disaccharides, with only 3.5% of glucans. Ginestra et al. [24] detected similar results, with a high level of glucose in dry cladodes and 36% of the total carbohydrate yield. However, the recorded differences could be related to the age of cladodes used in the studies and to agronomic and environmental factors. These polysaccharide fractions can be used both in the food industry for their technological characteristics and in the pharmaceutical industry for their bioactive properties. In fact, Di Lorenzo et al. [29] highlighted the ability of the mucilage fraction to repair dermal tissues through the creation of a physical barrier on the skin and deep hydration, thus promoting the skin repair processes. In addition, the OFI polysaccharides fraction can contribute to lipid and sugar metabolism regulation and could represent good substrates for intestinal microbiota. Moreover, the intake of OFI cladodes’ dietary fibre influences the glucose control and modulates the renal water and sodium level of type 2 diabetes patients [30].

#### 2.1.3. Ion Contents in *Opuntia ficus indica* Cladodes

Calcium (Ca) is the most abundant mineral in OFI, followed by sodium (Na), potassium (K) and magnesium (Mg), as shown in Table 3.

This trend was in disagreement with other authors, who found K as the most represented mineral [13,31]. However, independently to the trend, the Ca and Na levels found in this study were higher than those found by Hernández-Urbiola et al. [31] on the same vegetable matrix. These differences could probably be related to growing conditions such as salinity, and others chemical and physical parameters of soil; also, the spiny presence and maturity stage can affect the high Ca contents in OFI [13,21]. Regarding the anion content (nitrate, sulphate, chloride and oxalate), the most represented were sulphate and chloride, followed by nitrate and oxalate; the principal anti-nutritional elements identified in OFI. In particular, the nitrate concentration in plants’ food products is an important parameter for quality valuation and for consumer health [32]. As suggested by the European Food Safety Authority (EFSA, 2008), the current acceptable daily intake for this element is 3.7 mg/Kg of body weight. In our study, the nitrate contents in OFI were 580 mg/kg, which is in agreement with the results reported by Nerd and Nobel [33], and lower in respect to the nitrate limit imposed by the Commission Regulation (EU) No 1258/2011 for plants foods (3000–7000 mg/kg of fresh samples). Regarding oxalate, its intake plays a key role in secondary hyperoxaluria, a major risk factor for Ca-oxalate stone formation [34]. In the OFI samples analysed in this study, the oxalate level reported in Table 3 was lower with respect to those described by other authors, who reported a high variability of this element in relation to maturity stages [21].

### 2.2. Antioxidant Activity

The free radical-scavenging capacity of OFI powder was evaluated using DPPH and ABTS radical cation assays, and expressed as a TEAC (μmol Trolox/100 g OFI) value. Both relatively stable organic radicals have been widely used in the determination of the antioxidant activity of different vegetable matrices [35]. From the results presented in Table 4, the antioxidant capacity of OFI measured by DPPH**^∙^** showed the same behaviour as in the ABTS^+^ method, even with similar values; in particular, 740 and 775 μmol Trolox equivalent/100 g dw OFI, respectively.

Other authors have reported the antioxidant capacity of OFI cladodes using the same organic radicals. In particular, Andreu et al. [36] analyzed the antioxidant activity of different *Opuntia ficus indica* parts, such as cladodes and fruits, and found that fruits had a higher antioxidant power than cladodes. The latter were analyzed at two different ages: less than 1 year (young) and 2 years (old); the young cladodes were more active than the old ones. Furthermore, similar behavior between ABTS and DPPH methods was also recorded by the same authors, as well as reported in this study. Other authors, on cladode extract coming from different cultivars, obtained different data for both the methods used. In particular, De Santiago et al. [22] found ABTS values of 4500 μmol Trolox/100 g fresh weight and DPPH values of 20 μmol Trolox/100 g fresh weight, while Astello-Garcia et al. [20] reported a DPPH value of about 500 μmol Trolox/ g dry weight, more comparable to those reported in this manuscript. However, from the reported data, it is possible to underline that it is always difficult to compare the antioxidant activity values because of the lack of homogeneity in the results expression, and the differences among cultivars, physiological stage, and treatments for powder production [36].

### 2.3. In Vitro Digestion to Evaluate the Polyphenols and Minerals Bioaccessibility

#### 2.3.1. Polyphenol Bioaccessibility

Table 5 shows the results obtained after the three steps of the simulated gastrointestinal digestion of OFI cladodes. Some identified compounds, such as PI, EU and ferulic acids, are highly bioaccessible, with a percentage of up to 208% (piscidic acid I) when compared to their amount in the undigested sample (Table 1).

This effect could be explained by the partial binding of these polyphenols to the dietary fibre present in OFI cladodes that, during simulated digestion, are released in the small intestinal tract, increasing their bioaccessibility. In fact, polyphenols and dietary fibre have hydroxyl (-OH) and other functional groups that can create not-covalent bonds, such as hydrogen and Van der Waals. During the simulated digestion, the different pH and ionic strength of compartments could brake these linkages, with a consequent increase of polyphenols released and their bioaccessibility [37]. Additionally, possible isomerization reactions occurring among the stereoisomeric forms of piscidic and eucomic acids could also induce quantitative changes during gastrointestinal digestion [25]. Other published data [38] showed that the hydroxycinnamic acids, such as ferulic acid, linked to the wall cell in mild alkaline conditions similar to the human intestinal environment can be readily released, increasing their bioaccessibility (until 124%). Similarly, as reported by De Santiago et al. [25], the strong acidic condition occurring during the gastric phase could hydrolyse ferulic acid bound to pectin, increasing its bioaccessibility. Regarding the flavonoid class, their bioaccessibility ranged from 89% to 100% and no aglycone moieties were detected, probably because the salivary and pancreatic α-amylase activity was not able to break the β-glycosidic bond between the flavonoid aglycones and their glycosidic portion. The flavonoids’ de-glycosilation is due to the effect of cytosolic and membrane-bound β-glycosydases present in the brush border cells of the mammalian small intestine, or the action of gut microbiota [25]. Consequently, flavonoids undergo a degradation in respect to the phenolic acid, decreasing their relative abundance in the digested sample, to 15% with respect to the 28% of the total polyphenols before digestion. In any case, the high concentration of OFI cladodes’ polyphenols in the intestinal tract could be positive both for improving their intestinal absorption and for their interaction with gut microbiota, producing metabolites with additional biological properties.

#### 2.3.2. Cations’ Bioaccessibility

After the in vitro gastrointestinal digestion process, the major mineral released in the digested sample was Ca, followed by Mg and K. A similar level of Ca in digested samples was found in two different cladode cultivars (*Milpa Alta* and *Atlixco*) of OFI [39]. Instead, the main bioaccessible mineral was Mg, followed by K and Ca (Table 3). This low Ca bioaccessibility could be related to oxalate presence; in fact, this compound can combine with Ca and others minerals to form insoluble compounds, reducing their availability [34]. Similar results about Ca and K bioaccessibility were found in different leafy vegetables [40,41]. These elements are essential for human health, to maintain the electrolyte balance involved in many different bodily processes including bone growth, muscle and nerve functionality, and also to prevent osteoporosis diseases, as described by several authors [18,19,20,21,22,23,24,25,26,27,28,29,30,31,32,33,34,35,36,37,38,39,40,41,42]. The results reported here on the mineral composition of OFI cladodes suggest that consumtion of 5 g of OFI as natural supplements, or as an ingredient in foods, allows the intake of about 64 mg of Mg and 119 mg of Ca. Finally, the protocol applied does not allow the evaluation of Na bioaccessibility, because the solutions used in the in vitro digestion protocol show a high Na level and the blank correction cannot be performed. Other authors suggest that polyphenols, through their carboxylic groups, can form complexes with metal cations, thus interfering with their intestinal absorption. In particular, polyphenols can have a negative effect on the sodium bioavailability, but not on calcium or magnesium [43].

## 3. Materials and Methods

### 3.1. Reagents

For use as reagents, 6-Hydroxy-2, 5,7,8-tetramethylchromane-2-carboxylic acid (Trolox n 648471, Calbiochem San Diego, CA, USA,), 2,2′-Azino-bis (3-ethylbenzothiazoline-6-sulfonic acid (ABTS, A 1888, Sigma Aldrich, Taufkirchen, Germany)), 1,1-Diphenyl-2-picrylhydrazyl (DPPH, Sigma Aldrich), phosphate buffer solution (PBS), Ethanol (EtOH), Methanol (MtOH HPLC grade), Trifluoracetic acid, (TFA, Sigma Aldrich), nitric acid and acetic acid (HPLC gradient grade) were obtained from VWR Chemicals (Radnor, PA, USA). All other reagents were of analytical grade and the water was ultra-pure-grade. Phenolic standards: ferulic acid, tyrosol, isorhamnetin 3-O-rutinoside (narcissin) and kaempferol 3-O-glucoside were purchased from Extrasynthese (Genay, France). The in vitro digestion enzymes: α-amylase from *Bacillus* sp; pepsin, pancreatin, mucin, lipase from pigs; and bovine bile extract were obtained from Sigma-Aldrich (Milan, Italy).

### 3.2. Plant Materials

The plant material used in this study was the OFI cladode of the spineless cactus. *Opuntia* cladode powder was supplied by Cactus Bio Slim (Nopal Tunisie ZI route de Tala 1200 Kasserine Tunisie). This product was obtained from cladodes harvested in the area of Zelfane (Kasserine-Tunisia) from March to November 2017 (20 months of age), washed with pot water, cut into small pieces and dried at 45 °C, then ground and stored as reported by Santos-Zea et al. [44].

### 3.3. Extraction and Characterization of Phenolic Compound from OFI Cladodes

A total of 2 g of powder was extracted using a mixture of 50 mL methanol/water (80:20 *v*/*v*), then placed in an ultrasonic bath for 30 min, centrifuged and diluted two times. The resulting solutions were filtered using 0.45 µm cellulose regenerated filters and collected in an amber vial for further use. HPLC-DAD analysis was performed using an Agilent 1260 infinity system equipped with a 1260 binary pump, a 1260 Hip Degasser, a 1260 TCC thermostat, a 1260 Diode Array Detector and Agilent Open Lab Chem station Rev C 01.05 software (Agilent Technologies, Santa Clara, CA, USA). A reversed phase Luna C-18 (5 µm 4,6 × 250 mm) column (Phenomenex Torrance, CA, USA) was used for the chromatographic separation. The wavelengths used for the polyphenol detection were 280 nm (benzoic acids), 325 nm (phenilpropanoids) and 360 nm (flavonoids). The elution was performed using methanol (eluent A) and water–acetic acid 95:5 (eluent B). The gradient profile was 85–60% B (0–25 min), 60% B (25–30 min), 60–37% B (30–45 min), 37% B (45–47 min) and 37–0% B (47–52 min). The flow rate was 1 mL min^−1^. Samples were applied to the column using a 25 μL loop valve. The polyphenols were identified by retention time and spectra of the pure standards, when available. In particular, the piscidic and eucomic acids were expressed as tyrosol equivalents, ferulic derivative as ferulic acid; isorhamnetin and kaempferol derivatives were quantified as narcissin (isorhamnetin 3-O-rutinoside) and kaempferol 3-O-glucoside, respectively. The quantification was performed by the external standard method, using the following calibration curves: tyrosol 2–200 μg/mL, ferulic acid 0.2–10 μg/mL, kaempferol 3-O-glucoside 0.2–10 μg/mL and narcissin 1–100 μg/mL. Results were expressed as milligrams of each compound for 100 g of dry weight (d.w.) of the sample.

### 3.4. Dietary Fibre Determination

#### 3.4.1. Extraction of Soluble Fibre: Mucilage and Pectin Fractions

The mucilage fraction (MF) was recovered from the dehydrated OFI cladode powder following the procedure reported by Bayar et al. [45]. In particular, the sample was dissolved in water in a ratio of 1:10 *w*/*v* at a temperature of 25 °C and stirred for 90 min. Afterwards, the mixture was centrifuged at 4500 rpm for 15 min and the supernatant was precipitated with isopropanol at 4 °C overnight. Finally, the precipitate was washed with ethanol twice and left to dry at 50 °C to reach constant weight. The pectin fraction (PF) extraction was performed following the conventional methods reported by Bagherian et al. [27]. In particular, after the removal of the mucilage, the precipitate was suspended in acidified water (pH 2.8) and heated at 90 °C for 2 h. Afterwards, the mixture was submitted to centrifugation, concentration, precipitation, and washing and drying steps, following the method reported by Bayar et al. [26]. Finally, the pectin and mucilage fraction (PMF) was extracted following the same method reported for the pectin extraction but without mucilage removal. For the three fractions (mucilage, pectin, pectin–mucilage) the extraction yields (dry weight) were expressed as a percentage of the initial OFI powder.

#### 3.4.2. Polysaccharide Hydrolysis and Analysis

The main monosaccharides present in the MF, PF and PMF fractions were evaluated after acid hydrolysis by TFA, following the methods reported by Fengel & Wegener [46]. The hydrolysate mixtures were dried under vacuum and the residue was solubilized in 10 mL of H_2_O, and filtered. The carbohydrate analysis was performed with a HPLC Dionex DX500 system equipped with a GP50 gradient pump, an ED40 Electrochemical Detector and Dionex Peaknet 5.11 chromatographic Software (Dionex corporation, Sunnyvale, CA, USA). The monosaccharide separation was obtained using a Dionex CarboPac PA1 column (Dionex corporation, Sunnyvale, CA, USA) with a PA1 guard column; the mobile phases were sodium hydroxide (45 mM) and water at isocratic mode (10:90), the time course was 30 min at 35 °C and the flow rate was 1.0 mL/min. Meanwhile, the standard solutions of rhamnose, arabinose, galactose, glucose, mannose and xylose were used for the identification and quantification of the main monosaccharides.

#### 3.4.3. Estimation of Polysaccharides

The estimation of the homologous polymeric sugar content was performed using the concentration of the monomeric sugars released during the acid hydrolysis of the three polysaccharide fractions, as reported by Sluiter et al. [47]. The amount of each polysaccharide, as a percentage of dry matter, was determined as follows:

Glucan content (%) = (GH × 0.90/ Sample) × 100(1)

Galactan content (%) = (GaH × 0.90/ Sample) × 100(2)

Xylan content (%) = (XH × 0.88/ Sample) × 100(3)

Arabinan content (%) = (AH × 0.88 / Sample) × 100(4)

Mannan content (%) = (MH × 0.90/ Sample) × 100(5)

Where GH, XH, AH, GaH and MH represent glucose, xylose, arabinose, galactose, and mannose (g), using 0.90 and 0.88 as anhydrous correction conversion factors.

### 3.5. Mineral Determination

#### 3.5.1. Cation Content of OFI Cladodes

For the determination of Na, K, Mg and Ca contents, the protocol by D’Imperio et al. [40], with some modification of mineralization process, was used. Briefly, 0.3 g of OFI was accurately weighed in microwave digestion vessels, followed by the addition of 10 mL of 65% HNO_3_ and digestion in a closed-vessel microwave assisted digestion system (MARS 6, CEM Corporation, Matthews, NC, USA). The two step digestion procedure was performed as follows: 15 min to reach 200 °C and 10 min at 200 °C (constant T; power set at 900–1050 W; 800 psi). Blanks, HNO_3_ without the sample, were also prepared and digested using the same conditions, before the digested solutions were cooled and quantitatively transferred to 50 mL volumetric flasks, diluted to volume (50 mL) with ultrapure H_2_O (Milli-Q Millipore 18 M Ω cm^−1^, Burlington, Massachusetts, USA) and filtered using a 0.45 μm filter. The resulting solution was analysed by ion chromatography (Dionex DX120, Dionex Corporation, Sunnyvale, CA, USA) with a conductivity detector, using an IonPac CG12A guard column and an IonPac CS12A analytical column (Dionex Corporation, Sunnyvale, CA, USA) at 35 °C, flow 1 mL/min. The content of cations was calculated on the basis of the calibration curves previously obtained. Standard used: Multi Element IC Standard solution Fluka TraceCERT^®^, Supelco^®^ (Merck KGaA, Darmstadt, Germany), contains cation element. The limits of detection (LOD) and quantification (LOQ) were defined as the minimum amounts at which the analyte can be reliably detected and quantified. As follows, the values of LOD and LOQ of cations in µg/L were Na (0.239, 0.717), K (1.732, 5.196), Mg (1.371, 4.113) and Ca (0.806, 2.418). The accuracy and precision of the cation measurement procedures were verified by testing the certified reference standard 1573a-Tomato Leaf powder of the National Institute for Standards and Technology (NIST), with an element recovery average of 102 ± 5.5%.

#### 3.5.2. Anion Content of OFI cladodes

An ion exchange chromatography (Dionex DX120, Dionex Corporation, Sunnyvale, CA, USA) was performed with a conductivity detector, as reported by D’Imperio et al. [41]. For the determination of anion contents, 0.3 g OFI dry sample was treated with a solution of 3.5 mM (Na_2_CO_3_) and 1 mM (NaHCO_3_) for 30 min in orbital shaker, 50 rpm, at room temperature. After extraction, the samples were centrifuged (12,000× *g* seconds at room temperature) and filtered by using 0.45 µm (RC) followed by Dionex OnGuard IIP (ThermoScientific) in order to remove organic compounds (phenolic fraction of humic acids, tannic acids, lignins, anthocyanins, and azo dyes from sample matrices). The resulting solutions were analysed by ion chromatography (Dionex DX120, Dionex Corporation) with a conductivity detector, by using an IonPac AG14 precolumn and an IonPac AS14 separation column (Dionex Corporation). Samples were applied to the column using a 25 μL loop valve. For each extraction, three independent analyses were performed using 3.5 mM Na_2_CO_3_ and 1 mM NaHCO_3_, at a flow rate of 1 mL/min at 35 °C and 50 mA. The content of anions was calculated on the basis of the calibration curves previously obtained. Standard used: Multi Element IC Standard sol. IC-MAN-18 (6E), contains anions element in H_2_O (IC-MAN-18). The limits of detection (LOD) and quantification (LOQ) were defined as the minimum amounts at which the analyte could be reliably detected and quantified. As follows, the values of the LOD and LOQ of anions in mg/L: Cl (0.06, 0.195), NO_3_ (0.037, 0.122), SO_4_ (0.051, 0.153) and oxalate (0.072, 0.216).

### 3.6. Antioxidant Activity

Antioxidant activities were carried out following the methods of Liao et al. [48] for DPPH, and Re et al. [49] for ABTS.

#### 3.6.1. DPPH Assay

To evaluate the antioxidant activity of OFI phenolic extracts, the stable radical DPPH in 100% methanol as solvent was used, following the procedure of Liao et al. [48]. The reducing capability of OFI powder was quantified following the change of absorbance at 517 nm (UV–VIS Varian Cary 50 spectrophotometer, Varian Inc., Palo Alto, CA, USA) of DPPH solution after reacting with antioxidant compounds. The metanolic DPPH stock solution was properly diluted to an absorbance value of 1.1 before analysis. The OFI antioxidant capacity was calculated referring to Trolox Equivalent Antioxidant Capacity (TEAC) and the obtained results were expressed as μmol Trolox equivalent/100 g OFI, referring to the Trolox standard curve at concentrations ranging from 22 to 214 μg/mL. Six measurements for each sample were performed.

#### 3.6.2. ABTS Assay

The ABTS radical cation (ABTS∙⁺) was produced by reacting ABTS stock solution (7 mM in PBS) with 2.45 mM potassium persulfate in the dark for 12 to 16 h before use. In this mixture, ABTS and potassium persulfate react stochiometrically at a ratio of 1:0.5. The absorbance of ABTS⁺ solution was measured at 734 nm (UV-VIS Varian Cary 50 spectrophotometer) and adjusted at 0.700 with PBS buffer. An aliquot of 20 μL of OFI extract or Trolox was added to 2 mL of ABTS⁺ solution. The absorbance at 734 nm was recorded after 18 min. For the calibration curve, a methanol solution of Trolox, ranging from 7.8 to 150 μM, was used and the absorbance decrement was measured at 734 nm. The antioxidant capacity was expressed as μmol Trolox equivalent/100 g OFI.

### 3.7. In Vitro Digestion to Evaluate the Polyphenols and Mineral Bioaccessibility

The three stage in vitro digestion model was carried out as described by D’Antuono et al. [50] in order to evaluate the polyphenols and mineral bioaccessibility. This method simulates the oral, gastric and small intestinal physiological steps of the human digestion. The concentrations of enzymes used were reported as follows: α-amylase (370 mg/g OFI), mucin (2.5 mg/g OFI), uric acid (1.5 mg/g OFI), urea (20 mg/g OFI), 2 mL porcine pepsin solution (19 mg/mL in 0.1 M HCl), 2 mL mixed pancreatin (30 mg/mL) and lipase (15 mg/mL) in 0.1 M NaHCO_3_. Moreover, samples at a final volume of 50 mL were centrifuged (10,400× *g*, 4 °C, 1h) to recover the aqueous small intestinal digesta (DG). The DG were filtered and analysed by HPLC for polyphenols and ion analysis. Each experiment was performed in triplicate. The bioaccessibility, which represents the percentage of polyphenols, Ca, Mg and K released from the OFI in the DG fraction after GI digestion, was calculated as follows:

Bioaccessibility (%) = CF/CI × 100(6)

where CF is the polyphenols, Ca, Mg and K concentrations in the DG fraction and CI is the initial polyphenol, Ca, Mg and K concentrations in undigested dehydrated OFI.

## 4. Conclusions

The use of natural antioxidants is an increasing point of interest due to the continuous modification of the consumer’s choice. The results presented here suggest the possible use of natural OFI cladode powder for the enrichment of widely consumed foods, such as bread, pasta, biscuits, for functional food industrial application. The chemical characterization of OFI cladode powder allowed the identification of the main phenolic classes and subclasses, highlighting the presence of phenolic acids such as piscidic and eucomic acids, flavonoids, in particular isorhamnetins, and other minor compounds. The dietary fibres occurring in OFI can influence polyphenols’ bioaccessibility; in the simulated gastrointestinal conditions, the non-covalent bonds between polyphenols and dietary fibres were probably broken, with consequent polyphenol release in the upper and lower parts of digestive tract, increasing their availability for colonic microbiota action. It is noteworthy to underline the evaluation of magnesium bioaccessibility, not reported until now, that gives a further healthy connotation to this ingredient. Finally, considering the presence of dietary fibre in OFI cladode powder with a potential prebiotic effect, further studies will address the evaluation of the viability and metabolic activity of selected gut bacteria by an in vitro microbiota model.

## Figures and Tables

**Table 1:** Polyphenol content in *Opuntia ficus indica* cladodes.

	Compounds	mg/100 g OFI
1	Piscidic acid I	967.2 ± 35.9
2	Piscidic acid II	17.6 ± 5.2
3	Ferulic acid derivative I	6.5 ± 0.3
4	Eucomic acid	48.8 ± 2.2
5	Ferulic acid derivative II	3.6 ± 0.4
6	Kaempferol derivative I	6.8 ± 1.7
7	Isorhamnetin derivative I	254.4 ± 31.8
8	Isorhamnetin derivative II	54.4 ± 5.9
9	Kaempferol derivative II	1.7 ± 0.3
10	Isorhamnetin derivative III	9.3 ± 2.0
11	Isorhamnetin 3-O rutinoside (narcissin)	75.2 ± 8.8
	Total	1446.8 ± 67.0

**Table 2:** Extraction yields and total sugar content of three soluble fibre fractions from *Opuntia ficus indica* cladode powder (OFI).

	Yield (g/100 g OFI)	Total Sugar Content (g/100 g Fraction)
Mucilage	18.52 ± 1.76	9.19 ± 0.54
Pectin	7.11 ± 1.03	66.40 ± 4.11
Pectin/mucilage	12.06 ± 0.77	60.30 ± 5.75

**Table 3:** Anion and cation compositions in *Opuntia ficus indica* cladodes (OFI).

Ions	Concentration (mg/ g OFI)
*Cations*	
Sodium	19.18 ± 1.05
Potassium	16.84 ± 0.68
Magnesium	13.80 ± 1.37
Calcium	75.18 ± 1.62
*Anions*	
Nitrate	0.58 ± 0.06
Sulphate	6.26 ± 0.70
Chloride	7.52 ± 0.30
Oxalate	1.05 ± 0.06

**Table 4:** Antioxidant activity of *Opuntia ficus indica* cladodes (OFI) evaluated by in vitro assays. Results are expressed as mean ± standard deviation (*n* = 3).

	TEAC (μmol Trolox Equivalent/100 g OFI)
ABTS	775 ± 131
DPPH	740 ± 110

**Table 5:** Digested composition and bioaccessibility of polyphenols and cations from *Opuntia ficus indica* cladodes (OFI), after in vitro gastrointestinal digestion. Results are expressed as mean ± standard deviation (*n* = 3).

		Digested (mg/100 g OFI)	Bioaccessibility (%)
	*Polyphenols*		
1	Piscidic acid I	2018.9 ± 45.3	208.7
2	Piscidic acid II	21.4 ± 2.3	121.3
3	Ferulic acid derivative I	8.1 ± 0.2	123.8
4	Eucomic acid	56.7 ± 0.8	116.2
5	Ferulic acid derivative II	3.2 ± 0.1	88.6
6	Kaempferol derivative I	7 ± 0.2	102
7	Isorhamnetin derivative I	249.5 ± 7.4	98.1
8	Isorhamnetin derivative II	48.6 ± 1.4	89.4
9	Kaempferol derivative II	1.6 ± 0.1	96.5
10	Isorhamnetin derivative III	8.4 ± 0.3	90.7
11	Isorhamnetin 3-O rutinoside (narcissin)	66.9 ± 2.1	88.9
	Total polyphenols	2490.4 ± 55.6	172.1
	*Cations*		
	Potassium	883.7 ± 34.5	52.5
	Magnesium	1279.0 ± 75.1	92.7
	Calcium	2373.0 ± 106.9	31.6
